# Social Emotional Competence, Learning Outcomes, Emotional and Behavioral Difficulties of Preschool Children: Parent and Teacher Evaluations

**DOI:** 10.3389/fpsyg.2021.760782

**Published:** 2022-02-03

**Authors:** Baiba Martinsone, Inga Supe, Ieva Stokenberga, Ilze Damberga, Carmel Cefai, Liberato Camilleri, Paul Bartolo, Mollie Rose O’Riordan, Ilaria Grazzani

**Affiliations:** ^1^Department of Psychology, University of Latvia, Riga, Latvia; ^2^Department of Psychology, University of Malta, Msida, Malta; ^3^Department of Statistics and Operations Research, University of Malta, Msida, Malta; ^4^Centre for Resilience and Socio-Emotional Health, University of Malta, Msida, Malta; ^5^Department of Human Science for Education “R. Massa,” University of Milano-Bicocca, Milan, Italy

**Keywords:** social emotional competence, emotional and behavioral difficulties, learning outcomes, preschool children, socioeconomic status, multi-informant assessment

## Abstract

This paper addresses the role of social emotional competence in the emotional and behavioral problems and learning outcomes of preschool children based on their parents’ and teachers’ evaluations. In this study, we compared the perceptions of teachers and parents when evaluating the same child using the multi-informant assessment. First, the associations and differences between both the informant evaluations were investigated. Second, the correlation of the social emotional competence and emotional, and behavioral difficulties among preschool children was analyzed, separately addressing their parents’ and teachers’ evaluations. Third, the role of the preschool children’s social emotional competence in their emotional and behavioral problems, and learning outcomes was investigated building the regression and mediation models. The sample consisted of 507 preschool children (3–6 years, mean age 4.85 years, SD 0.82) and their parents and teachers. Both informants completed the Strengths and Difficulties Questionnaire and Social Skills Improvement System Social-Emotional Learning Brief Scales, and teachers reported on each child’s learning outcomes (by completing a three-item Learning outcomes measure). When comparing both informants’ evaluations, positive associations were found between teacher and parental evaluations of prosocial behavior and emotional, and behavioral difficulties of preschool children, as well as self-management. Parents evaluated their children higher than teachers in conduct problems, hyperactivity, prosocial behavior, and total difficulty, while teachers evaluated children higher than parents in social emotional competence. According to teachers, the social emotional competence of preschool children was negatively correlated to all difficulty scales, and positively related to the prosocial behavior scale. The demographic variables, as well as parental socioeconomic status and children’s belonging to a vulnerable group were not found to be significantly associated with the preschool children’s learning outcomes. However, social emotional competence remains a significant variable in teacher-rated learning outcomes of preschool children even if sociodemographic variables are controlled. Our findings indicate that a higher level of social emotional competence and lower levels of social, emotional, and behavioral difficulties are related to a higher preschoolers’ academic learning in their teachers’ evaluation. This suggests the importance of early facilitation of social emotional competence as a key factor for academic success and more positive behavioral outcomes.

## Introduction

The social emotional competence of children has been a central topic for both practitioners and researchers in the past several decades. It has been generally recognized that the key intrapersonal and interpersonal skills that build social emotional competence include self-awareness, self-management, social awareness, relationship skills, and responsible decision-making. These skills are important in different aspects of life (e.g., Zins et al., 2004; CASEL, 2020), and they are the best traits to invest in to develop well-being and mental health, moral reasoning, academic performance, and achievement motivation (e.g., Durlak et al., 2011). The process of acquiring and applying social emotional competence (consisting of knowledge, skills, and attitudes) is recognized as an integral part of education and human development and is defined as social emotional learning (CASEL, 2020). In a longitudinal study, Jones et al. (2015) reported that social emotional learning in early years was related to positive outcomes in different aspects of adulthood, such as mental health and well-being, relationships, careers, and decreased likelihood of delinquency and criminality.

Social and emotional development during preschool is rapid; hence, the impact of social emotional learning is higher during this stage as children’s behavioral patterns are still developing (Fisak et al., 2011). Different social emotional skills are important in diverse aspects of even very young children’s life. The number of studies on the social emotional competence of young children is growing (e.g., Denham et al., 2013; Merry, 2015). Improved social emotional skills in early childhood are related to more successful development of cognitive and social skills (Camilli et al., 2010; Kautz et al., 2014). In a study, 2–3-year-old children (Brazzelli et al., 2021) demonstrated significantly more prosocial behavior (helping, sharing, and comforting) after a social emotional learning. Prosocial behavior plays a significant role in peer acceptance, and relationship building and maintenance (Spinrad and Eisenberg, 2017). Those 5–6-year-olds with better self-management are recognized as being more able to adapt to the requirements of preschool (Rimm-Kaufman et al., 2005). Children with higher social emotional competence in preschool build and maintain more successful relationships with parents and teachers at the start of school and have more positive interactions with peers (McCabe and Altamura, 2011; Blair and Raver, 2015).

It is known that higher social emotional competence is associated with decreased behavioral and emotional problems. Preschool-aged children who have been seen by teachers as having better behavior management skills and better self-regulation show higher engagement in preschool activities. Similarly, there is evidence that low social emotional competence is related to the manifestation of problematic behavior and emotional difficulties, as well as relationship problems (e.g., Durlak et al., 2011; Sklad et al., 2012). Children, even toddlers (Ornaghi et al., 2017) who rarely display prosocial behavior manifest various behavioral problems, particularly aggression, and can also experience peer rejection at school. There is a significant correlation between preschool-aged children’s hyperactivity and behavioral problems at school. Aggressive and oppositional behavior in preschool caused by poor behavioral self-regulation is associated with emotional lability, lower social skills, lower academic achievement, and less participation in the learning process (Fantuzzo et al., 2003; Blair and Diamond, 2008; Domínguez and Greenfield, 2009).

To evaluate children’s behavior more objectively, evidence suggests both direct observations (e.g., Pellegrini, 2001) and obtaining multi-informant data. Combining parent and teacher evaluations allows a more accurate estimate of the difficulties among preschool-aged children (Fält et al., 2018). Additionally, preschool children cannot complete questionnaires by themselves; thus, evaluations from both parents and teachers can be used for inter-rater reliability. For this reason, the multi-informant approach is recognized as effective in psychological assessments including populations of young children (Huber et al., 2019; Anthony et al., 2020). Moreover, Evans (2015) claims that assessment of the social emotional development of young children should be done using multiple methods and multiple-informant sources.

In terms of their evaluations of children’s prosocial behavior and social and emotional difficulties, teachers and parents provide distinct perspectives based on different contexts. In their large-scale meta-analysis, Stone et al. (2010) found a modest positive correlation between teachers’ and parents’ evaluations of children’s prosocial behavior and social and emotional difficulties on the Strengths and Difficulties Questionnaire (SDQ). In general, parents’ and teachers’ evaluations show mutual agreement; however, the differences that do exist between teachers’ and parents’ assessments of children’s behavioral problems and prosocial behaviors could be explained by the fact that teachers see children in a social context with other children (Stone et al., 2010) more often than parents do. The authors show that most studies exploring strengths and difficulties are conducted with school-aged children, and more evidence is needed about preschool-aged children, especially using multi-informant data sources (teachers and parents).

Simultaneously, children come to preschool from different social environments; some are at risk of social exclusion, which could affect their behavior (Thompson, 2014). It is also possible that children with better developed social emotional competence will be seen in a more positive light, as they elicit more positive attitudes from teachers (Eisenhower et al., 2007).

There is also a growing recognition of the role of social emotional competence in academic learning (Panadero and Alonso-Tapia, 2013; Panadero et al., 2017). Preschool children’s awareness of their own emotions and those of others, and their ability to adapt and follow instructions are important factors that explain their readiness for school (Anthony et al., 2020). Higher social emotional competence at an early age is associated with better ability to deal with academic difficulties (Denham et al., 2014; Bierman et al., 2016). Preschool children’s regulation of emotions, impulses, and attention have an important role in their subsequent school achievement to the same extent as their cognitive abilities and reading skills do (Raver and Knitzer, 2002). It has also been reported that preschool children’s positive engagement with teachers is associated with better self-regulation and capacity to remain task-oriented (Williford et al., 2013). Children’s capability to create and sustain a good quality relationship with their teachers is recognized as an important variable in preschool children’s academic readiness and early school adjustment (Palermo et al., 2007), including those in academically at-risk groups (e.g., Liew et al., 2010). On the contrary, a conflicting relationship is associated with poorer academic achievement and higher school avoidance among students (e.g., Ladd and Burgess, 2001). Previous studies have also reported that students’ adaptation, emotion regulation, and the ability to maintain friendly relationships with others are key factors in higher academic motivation, increased capacity to invest their own resources within the educational process, and sustained positive attitudes toward school (e.g., Elias et al., 1997; Raver et al., 2007; Merrell and Guelder, 2010; Durlak et al., 2011; CASEL, 2012; Weaver and Wilding, 2013).

In preschool, there is a challenge to evaluate children’s learning outcomes, since it is not possible to base the evaluation on grades as it is in samples of school children (e.g., OECD, 2021). A well-recognized and recommended approach is direct observations (e.g., Pellegrini, 2001; Nock and Kurtz, 2005); however, it is more applicable to research of children’s behavior. In the current research, we evaluated learning outcomes based on teachers’ views of students’ academic progress in terms of academic motivation, learning engagement and academic performance. To reach inter-rater agreement, the children’s two teachers were asked to reach a consensus and assess all children in the three aspects of motivation, engagement, and academic performance. This approach is in line with the multi-informant approach, where gathering information from various sources provides the opportunity to obtain a more objective evaluation of the situation.

Based on the presented review we aimed to explore the relationships among social emotional competence, social, emotional, and behavioral difficulties, and teacher-reported learning outcomes among preschool children. We also compared the perceptions of teachers and parents when evaluating the same child.

The following research questions were posed:

Q1: What is both associations and differences in teachers and parents’ evaluations on preschool children’s social emotional competence and social, emotional, and behavioral difficulties?
Q2: Is preschool children’s social emotional competence significantly related to their social, emotional, and behavioral difficulties according to the respective evaluations of teachers and parents?
Q3: To what extent, if any, social, emotional, and behavioral difficulties and social emotional competence explain teacher-reported learning outcomes of preschool children, when controlling for parents’ education, occupation, and child’s vulnerability?
Q4: Does social emotional competence mediate the relationship between social, emotional, and behavioral difficulties and teacher-reported learning outcomes in preschool children?

## Methodology

### Research Context and Participants

This research is a part of the ERASMUS + funded European international project “Promoting mental health at schools,” aiming to develop evidence-based programs and innovative policies focused on students’ mental health. This project involves seven European countries: Italy, Latvia, Portugal, Croatia, Romania, Greece, and Malta. The program was developed to promote the social emotional learning and resilience of school children while preventing emotional and behavioral problems. To evaluate the efficacy of the program, a quasi-experimental study design with experimental and waiting-list control groups was used. The target age groups of the project were school children from early years to high school (3–6, 8–10, 11–13, and 15–16 years). Data from the pre-test results of the youngest age group (preschool) of the Latvian sample were used in the present study. There were seven preschools from Latvia included in the project; all the preschools were of Sigulda region. Owing to the pre-test condition, data from all participants were collected and analyzed as a whole to explore associations among the target research variables, using the multi-informant approach.

The sample consisted of 507 preschool children (3–6 years, mean age 4.85 years, *SD* 0.82). Both genders were represented equally (boys 50.5 and girls 49.5%). The majority of these children (93.3%) did not have special educational needs and did not belong to any group at risk of social exclusion (minority or migrant). In terms of education, 58.3% of the parents completed tertiary education, 24.2% completed post-secondary education, and the remaining 17.5% completed, at most, secondary education. In terms of employment, 27.2% of the parents had a professional, managerial, or administrative job; 40% were skilled workers; 20.6% did semi-skilled or unskilled work; 11.2% were house carers; and the remaining 1% were unemployed or on state income. Informants were the children’s respective parents and teachers. Participant demographics are included in Table 1.

**TABLE 1:** Demographic characteristics of study participants (*n* = 507).

		Frequency	Percentage
Gender of preschool children	Male	256	50.5%
	Female	251	49.5%
Child requires special	Yes	34	6.7%
educational needs or belongs to a group at risk of social exclusion	No	473	93.3%
Child’s age	3 Years	6	1.2%
	4 Years	200	39.4%
	5 Years	169	33.3%
	6 Years	132	26.0%
Parent education	Post-graduate	106	21.0%
	Tertiary	189	37.3%
	Post-secondary	123	24.2%
	Primary/secondary	89	17.5%
Parent occupation	Professional	44	8.7%
	Managerial/administrative	94	18.5%
	Skilled/technical worker	203	40.0%
	Semi-skilled worker	82	16.2%
	Manual/unskilled worker	22	4.4%
	House carer	57	11.2%
	State income/unemployed	5	1.0%

### Procedure

The data collection took place in the beginning of a school year, respectively, in October, 2020. The participants were recruited through informative campaigns organized by the researchers in the seven preschools. Initially, informative letters with the invitation to participate in the survey were sent to both teachers and parents of all children of the specific age group. Then teacher and parent surveys and the informed consent forms were included in envelopes and delivered by researchers to every preschool. The teachers distributed parental questionnaires among the parents of the children in their groups. Since there are two teachers working in every preschool group, during the filling of the teacher survey, they were asked to reach agreement if their evaluation of the particular child differed. Before they completed the survey, all respondents gave informed consent to participate. Parents filled their surveys at home and returned them in closed envelopes to the group teacher of their child. The closed envelopes were collected by the researchers. Finally, the researchers opened the envelopes, checked if the informed consent was signed, and then inputted all answers in the electronic data file. To enable comparisons between teachers’ and parents’ evaluations, a code was generated for each child such that questionnaires completed by the teacher and parent of the same child had the same code. Only those preschool children who were assessed by both teachers and parents were included in the study. Initially, 591 informative letters were sent and the response rate was 95%. The initial sample comprised of 562 preschool children; however, children, who were assessed by teacher only and by parent only, or whose code was entered incorrectly, were excluded from the study. Thus, the final sample comprised of 507 preschool children. For the few missing data, we used an imputation process, where the missing values were replaced by the median item score.

The Ethics Committee for Humanities and Social Sciences Research Involving Human Participants of the University of Latvia granted permission for the research on 12 December, 2019.

### Measures

Teachers and parents of the preschool children were asked to fill down The Strengths and Difficulties Questionnaire (SDQ, Goodman, 1997) and the Social Skills Improvement System Social-Emotional Learning Brief Scales (SSIS SEL; Elliott et al., 2020a,b). Teachers were also asked to complete the brief Questionnaire of learning outcomes.

The SDQ (Goodman, 1997) evaluates the mental health of young children and adolescents. The difficulty scoring algorithm comprises four scales of five items each that measure emotional, conduct, hyperactivity, and peer problems. Each item is measured on a 3-point Likert scale ranging from 0 (not true) to 2 (certainly true), and the score for each scale ranges from 0 to 10. The total difficulty score, which combines the four difficulty scales, ranges from 0 to 40, where larger scores correspond to more social, emotional, and behavioral difficulties. In addition, the prosocial scoring algorithm comprises one scale of five items, where each item is measured on a 3-point Likert scale ranging from 0 (not true) to 2 (certainly true). The prosocial scale ranges from 0 to 10; larger scores indicate higher intent to benefit others. The factor structure of the SDQ was investigated in several studies (e.g., Goodman, 2001; Dickey and Blumberg, 2004; Cefai et al., 2009; Goodman et al., 2010; Niclasen, 2013) and in the current study, the five-factor model was used.

The SSIS SEL Brief Scales (Elliott et al., 2020a,b) Parent K-12 form and Teacher K-12 form evaluate children’s well-being and social emotional learning. The social emotional competence scoring algorithm comprises five scales of four items each that measure self-awareness, self-management, social awareness, relationship skills, and responsible decision-making. Each item is measured on a 4-point Likert scale ranging from 0 (never) to 3 (almost always), and the score for each scale ranges from 0 to 12. The SSIS SEL score, which combines the five social emotional learning scales, ranges from 0 to 60, where larger scores correspond to better management of emotions and achievement of personal goals, enhanced empathy for others, more supportive relationships, and more responsible and caring decisions. The 20-items SSIS SEL Brief scales for teachers and parents were generated from the 51-item SSIS SEL Rating Forms (Gresham and Elliott, 2017) using Item Response Theory (IRT). Moreover, reliability evidence for the SEL composite scores included Cronbach’s alphas (Teacher 0.95; Parent 0.91; Student 0.94) and inter-rater reliability (Teacher 0.65; Parent 0.61).

The SDQ teacher and parent forms were already validated in Latvian language, but SSIS SEL Brief scales was translated according to process recommended by Beaton et al. (2000) including translation, review, back-translation, and final agreement of the experts committee to reach semantic and conceptual equivalence with the original measure.

The teacher questionnaire included one more—the Learning outcomes measure. The children’s learning outcomes (consisting of three items regarding the academic motivation, learning engagement and academic performance) were measured by asking teachers to rate directly three aspects on a 5-point Likert scale ranging from 0 (very weak) to 4 (very good). The questions were as follows: How would you rate the student’s learning in terms of (1) academic motivation, (2) engagement in learning process and (3) academic performance. The pairwise correlations between these three questions all exceeded 0.84, so it was decided to combine these three questions in a single scale (learning outcomes of preschool children). The new scale, which was generated by summing the rating scores, ranges from 0 to 12, where the larger the score, the higher is the learning outcome. This measure was developed especially for this research, since there is no possibility to measure learning outcomes in a preschool population by grades as it is possible for school-age children (e.g., OECD, 2021).

In Latvian preschools, there are two teachers working in every group. Thus, every questionnaire on children’s social emotional competence, difficulties, and learning outcomes was completed by both teachers together. The teachers were asked to discuss discrepancies in their opinions and reach consensus when completing the questionnaire about every child. Consequently, we reached an inter-rater agreement to add strength to this indirect measure of learning outcomes.

Teachers’ questionnaire also included demographic questions about the children’s age and gender, and social exclusion. The parent questionnaire additionally asked for information about the parents’ education and occupation levels.

The reliability of all instruments of this study was checked, finding that the items measuring these scales have satisfactory internal consistency. Both composite scales combining the SDQ difficulty subscales and the SSIS-SEL subscales also have satisfactory internal consistency. Moreover, a three-item preschool children’s Learning outcomes measure, answered by teachers, demonstrated excellent internal consistency (see Table 2).

**TABLE 2:** Internal consistency of each scale and composite scales of SDQ, SSIS SEL based on preschool children’s (*n* = 507) teacher and parent evaluations, and teacher evaluations of learning outcomes on a three-item scale.

	Scales	Cronbach’s alpha	
		Teachers’ evaluation	Parents’ evaluation
SDQ	Emotional problems	0.783	0.716
	Conduct problems	0.727	0.732
	Hyperactivity	0.869	0.803
	Peer problems	0.713	0.725
	Prosocial behavior	0.780	0.754
	Total difficulty*	0.779	0.751

SSIS SEL	Self-awareness	0.760	0.727
	Self-management	0.754	0.733
	Social awareness	0.814	0.789
	Relationship skills	0.766	0.764
	Responsible decision making	0.849	0.766
	Social emotional competence**	0.798	0.754

	Learning outcomes	0.951	

*Refers to composite scale combining the SDQ difficulty subscales.

**Refers to composite scale combining the SSIS-SEL subscales.

### Data Analysis

Several statistical tests and models were used to analyze the data. Cronbach’s alpha coefficient was computed to assess the internal consistency of the items measuring each scale; values exceeding 0.7 indicate satisfactory internal consistency. The Kolmogorov-Smirnov test was used to investigate the normality assumption of each scale distribution. Non-parametric tests were used to analyze the data further, since all the scale distributions were skewed and violated the normality assumption. The Spearman correlation test was used to measure the strength of the associations between teachers’ and parents’ evaluations for each scale. Moreover, this test was also used to separately measure the strength of the associations between the SDQ and SSIS SEL scales for teachers’ and parents’ evaluations. The Wilcoxon signed ranks test was used to compare median scores of teachers’ and parents’ evaluations for each scale. A regression model was fitted to relate academic learning to six variables, including social emotional competence, social, emotional and behavioral difficulties, student vulnerability status, student gender, parental education and occupation. Finally, a mediation model was fitted to identify and explain the mechanism that underlies the relationship between academic learning (dependent variable) and total difficulty (independent variable) via social emotional competence (mediator). Baron and Kenny (1986) describe a four-step procedure to test for mediation by fitting three regression models. The Sobel test is then used to determine whether the indirect effect between academic learning and total difficulty via social emotional competence is significant.

## Results

Addressing the correlations between teacher and parental evaluations of preschool children’s social emotional competence and social, emotional, and behavioral difficulties, the scores provided by teachers for all the SDQ subscales (both the four difficulties subscales and the one prosocial behavior subscale), and only one of the SSIS SEL scale of self-management were positively and significantly related to the scores provided by parents (see Table 3). However, for the remaining SSIS SEL scales (self-awareness, social awareness, relationship skills, and responsible decision-making), the associations were weak and not significant.

**TABLE 3:** Association between teachers’ and parents’ evaluations of preschool children (*n* = 507): SDQ and SSIS SEL.

Scales		Spearman’s correlation
SDQ	Emotional problems	0.129**
	Conduct problems	0.294***
	Hyperactivity	0.373***
	Peer problems	0.239***
	Prosocial behavior	0.226***
	Total difficulty (composite scale)	0.305***
SSIS SEL	Self-awareness	0.040
	Self-management	0.163***
	Social awareness	0.030
	Relationship skills	0.072
	Responsible decision making	0.054
	Social emotional competence (composite scale)	0.125**

**p < 0.01; ***p < 0.001.

The differences between the median scores provided by preschool children’s teachers and parents were compared and tested (see Table 4). The results showed that in SSIS-SEL scales teachers rated children’s self-awareness, self-management, social awareness, responsible decision-making, and social emotional competence higher than did their parents. However, there was no discrepancy between teachers’ and parents’ evaluations of children’s relationship skills, evaluated by SSIS-SEL brief scales. It was also found that in SDQ parents tended to rate children’s prosocial behavior, conduct and hyperactivity problems, and total difficulty higher than their teachers did, that is, parents tend to inflate good and bad behavior compared to teachers. There was no discrepancy in SDQ scores between teachers and parents when evaluating children’s emotional and peer problems (i.e., internalizing behavior).

**TABLE 4:** Median and interquartile range of teachers’ and parents’ evaluations of preschool children (*n* = 507) on SDQ and SSIS SEL Scales.

SDQ		Median	IQ range	*P*-value	Size effect
Emotional problems	Teachers	1	0–3	0.030	0.030
	Parents	1	0–3		
Conduct problems	Teachers	1	0–3	0.231	–0.231
	Parents	2	1–3		
Hyperactivity problems	Teachers	3	1–6	0.000	–0.161
	Parents	4	2–6		
Peer problems	Teachers	2	0–4	0.610	0.023
	Parents	2	1–3		
Prosocial behavior	Teachers	7	5–9	0.000	–0.330
	Parents	8	6–9		
Total difficulty*	Teachers	8	4–13	0.007	–0.122
	Parents	9	7–13		

**SSIS SEL**		**Median**	**IQ range**	***P*-value**	**Size effect**

Self-awareness	Teachers	8	6–9	0.000	0.214
	Parents	7	5–8		
Self-management	Teachers	8	6–9	0.000	0.641
	Parents	6	5–7		
Social awareness	Teachers	8	6–9	0.003	0.135
	Parents	7	6–9		
Relationship skills	Teachers	8	7–10	0.930	0.004
	Parents	8	7–10		
Responsible decision making	Teachers	8	6–10	0.000	0.217
	Parents	7	6–9		
Social emotional competence**	Teachers	39	32–47	0.000	0.300
	Parents	35	31–40		

*Refers to composite scale combining the SDQ difficulty subscales.

**Refers to composite scale combining the SSIS-SEL subscales.

Regarding the second research question, the correlation of preschool children’ s social emotional competence and strengths, and difficulties was calculated separately in both informants’ evaluations (see Table 5). The teachers’ evaluations showed that all SSIS SEL scales were negatively and significantly related to the SDQ difficulty scales, and positively and significantly related to the SDQ prosocial behavior scale. Similar results were obtained when analyzing parents’ evaluations of their children’s all four SDQ difficulties scales and the prosocial behavior scale, and both SSIS SEL self-awareness and self-management; however, the associations between the SDQ scales and other SSIS SEL scales (social awareness, relationship skills, and responsible decision-making) were weak and not significant, except the negative correlation between the SDQ emotional problems and SSIS SEL measured relationship skills.

**TABLE 5:** (A) Correlations measuring associations between SDQ and SSIS SEL scales (teachers’ evaluations) in preschool children’s sample (*n* = 507). (B) Correlations measuring associations between SDQ and SSIS SEL scales (parents’ evaluations) in preschool children’s sample (*n* = 507).

SSIS SEL scales SDQ	Self-awareness	Self-management	Social awareness	Relationship skills	Responsible decision-making
**(A)**					
Emotional problems	−0.509***	−0.340***	−0.329***	−0.427***	−0.387***
Conduct problems	−0.473***	−0.628***	−0.511***	−0.523***	−0.605***
Hyperactivity	−0.651***	−0.761***	−0.576***	−0.614***	−0.705***
Peer problems	−0.606***	−0.450***	−0.532***	−0.654***	−0.527***
Prosocial behavior	0.664***	0.584***	0.754***	0.697***	0.694***
**(B)**					
Emotional problems	−0.268***	−0.321***	–0.001	−0.100**	–0.007
Conduct problems	−0.323***	−0.553***	–0.050	–0.070	–0.073
Hyperactivity	−0.310***	−0.527***	–0.001	–0.039	–0.039
Peer problems	−0.291***	−0.240***	–0.024	–0.029	–0.044
Prosocial behavior	0.445***	0.405***	0.048	0.045	0.046

**p < 0.01; ***p < 0.001.

To investigate the association of socioeconomic status (SES) with preschool children’s social emotional competence, strengths and difficulties, a scale was generated by combining parental education and occupation levels. The SES scale was then categorized into three levels (low, medium, high). Table 6 shows that higher SES is related to higher children’s social emotional competence and strengths, and reduces difficulties.

**TABLE 6:** Medians and interquartile ranges of SSIS SEL and SDQ scale scores of preschool children (*n* = 507) grouped by parents reported socioeconomic status (SES).

SDQ	SES	Median	IQ range	*P*-value	Size effect
Emotional problems	High	0.5	0–2.5	0.020	0.072
	Medium	1	0–3		
	Low	1.5	0–3.5		
Conduct problems	High	1	0.5–2	0.001	0.094
	Medium	2	1–3.5		
	Low	3	1.5–4		
Hyperactivity problems	High	3	1–4.5	0.001	0.099
	Medium	4	2–6		
	Low	5	3–7		
Peer problems	High	1	0.5–2	0.005	0.085
	Medium	2	1–3.5		
	Low	3	1.5–4		
Prosocial behavior	High	8	6–9	0.972	0.006
	Medium	8	6–9		
	Low	8	6–9		
Total difficulty*	High	6	4–10	0.000	0.126
	Medium	9	7–13		
	Low	12	10–16		

**SSIS SEL scales**	**SES**	**Median**	**IQ range**	***P*-value**	**Size effect**

Self-awareness	High	7.5	6–9	0.020	0.072
	Medium	7	5.5–8.5		
	Low	6.5	5–8		
Self-management	High	7	5.5–8.5	0.115	0.054
	Medium	6.5	5–8		
	Low	6.5	5–8		
Social awareness	High	7	6–9	0.915	0.011
	Medium	7	5.5–8.5		
	Low	7	5–8		
Relationship skills	High	8	6.5–9.5	0.324	0.039
	Medium	8	6.5–9.5		
	Low	7.5	6–9		
Responsible decision making	High	7.5	6–9	0.057	0.062
	Medium	7	5.5–8.5		
	Low	6.5	5–8		
Social emotional competence**	High	38	34–43	0.123	0.053
	Medium	35	31–40		
	Low	33	28–38		

*Refers to composite scale combining the SDQ difficulty subscales.

**Refers to composite scale combining the SSIS-SEL subscales.

To address the third research question, the regression model was built for dependent variable learning outcomes to six variables, including: social emotional competence; social, emotional, and behavioral difficulties; student vulnerability status; student gender; parental education; and occupation (see Table 7).

**TABLE 7:** Regression analysis for learning outcomes of preschool children (dependent variable) with parents’ education, occupation, child’s vulnerability, social emotional competence and, social, emotional, and behavioral difficulties as independent variables (*n* = 507).

Parameter	B	Std. Error	t	*P*-value
Intercept	3.783	1.111	3.406	0.001
Social emotional competence	0.113	0.013	8.512	0.000
Social, emotional, behavioral difficulties	–0.091	0.020	–4.574	0.000
Pupil at risk = Yes	–0.460	0.343	–1.341	0.181
Pupil gender = Boy (1), Girl (0)	–0.197	0.174	–1.131	0.259
Parent education = Post-graduate	0.134	0.322	0.415	0.678
Parent education = Graduate	0.176	0.275	0.641	0.522
Parent education = Post-secondary	–0.002	0.282	–0.008	0.994
Parent education = Secondary/primary	0			
Parent occupation = Professional	0.973	0.868	1.121	0.263
Parent occupation = Managerial/Administrative	0.977	0.841	1.161	0.246
Parent occupation = Skilled/Technical worker	0.824	0.827	0.996	0.320
Parent occupation = Semi-skilled worker	0.536	0.851	0.630	0.529
Parent occupation = Manual/unskilled worker	0.856	0.981	0.872	0.383
Parent occupation = House carer	0.763	0.856	0.892	0.373
Parent occupation = State income/unemployed	0	.	.	.

*R-squared = 0.518.*

This model, which explains 51.8% of the total variation in the learning outcomes scores, identifies social emotional competence and social, emotional, and behavioral problems as the two factors that are associated with learning outcomes significantly. The other four were not found to be significant explanatory variables.

The results show that higher level of social emotional competence and lower levels of social, emotional, and behavioral difficulties is associated to a better academic learning. In fact, for every 1 unit increase in social emotional competence, the learning outcomes score is expected to increase by 0.113; while for every unit increase in behavioral problems, the academic learning score is expected to decrease by 0.091. Although student gender, risk status, parental education, and occupation are not significantly associated with the students’ learning outcomes, there are indications that boys with special educational needs are at risk of social exclusion, and those whose parents have low level of education and have a low employment status, tend to score lower on academic learning than their peers.

**FIGURE 1:**
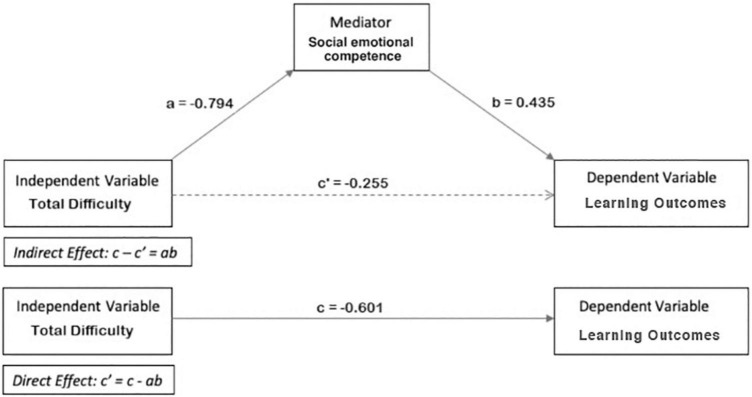
Direct, indirect and total effect in a mediation model.

Figure 1 displays the results of the mediation model, which was built to answer the last question of this study. The first model, which relates academic learning to SDQ total difficulty, yields a total effect size of c = –0.641. The second model, which relates SSIS SEL social emotional competence to SDQ total difficulty, yields the effect size of a = –0.794. The third model, which relates academic learning to both SDQ total difficulty and SSIS SEL social emotional competence, yields the sizes of the effects b = 0.490 and c‘ = –0.252. The Sobel test shows that the indirect effect between academic learning and SDQ total difficulty via SSIS SEL social emotional competence (ab = –0.389) and the direct effect (c‘ = –0.252) are both significant. This implies partial mediation, which indicates that both higher levels of social emotional competencies and lower behavioral difficulties are essential to enhance academic learning in preschool children.

## Discussion

In this study, we addressed the association among preschool children’s social emotional competence, learning outcomes, and social, emotional, and behavioral difficulties. To gain an in-depth understanding of the interrelations among these variables, we used a multi-informant approach, i.e., parents’ and teachers’ reports. Moreover, the teachers’ reports were based on the two teachers’ agreement. We were also interested in the extent of the similarity between both informants’ reports, while also considering demographic variables such as parental education and occupation level and vulnerable group status of a child. These findings will be discussed in turn.

### Associations and Differences in Teachers and Parents’ Evaluations on Preschool Children’s Social Emotional Competence and Social, Emotional, and Behavioral Difficulties

It was assumed that there would be a moderate correlation between parents’ and teachers’ reports of the emotional and behavioral difficulties and social emotional competence of preschool children in this sample. We found that teachers’ evaluations of children’s difficulties and prosocial behavior as well as self-management were positively and significantly related to those of the parents. However, there were no associations between parental and teacher evaluations of the children’s social emotional competence on aspects such as self- and social-awareness, relationship skills, and responsible decision making. Moreover, other research confirms that teachers and parents evaluate children’s behavior similarly (e.g., Cai et al., 2004), but social competence differently (Heyman et al., 2018). It could be interpreted that social emotional competence manifests in different settings differently. When comparing both informant reports, there was no discrepancy between teachers and parents when evaluating such difficulties as emotional and peer problems, hyperactivity and conduct problems, as well as children’s prosocial behavior. It was also found that the scores provided by teachers for one domain of social emotional competence (self-management) were positively and significantly related to the scores provided by parents. This result is in line with a previous meta-analysis showing that multi-informant reports are positively associated (Stone et al., 2010). However, there was a discrepancy in parental and teacher evaluations of preschool children’s such aspects of the social emotional competence as self-awareness, social awareness, relationship skills, and responsible decision making. Preschool teachers reported higher scores for their students’ self-awareness, social awareness, and responsible decision-making skills than parents. Moreover, the parents in this sample claimed that their children have more conduct and hyperactivity problems than the teachers did. The parents also reported that their children were more prosocial than the teachers did. Moreover, other studies claim that parents may be more confronted with their child’s everyday behavior management issues and the maintenance of rules. In a cross-cultural study in 13 countries, Rescorla et al. (2012) found that parents reported significantly more behavioral problems in their children than did teachers. The authors explained this finding by the fact that parents spend more time with their children and are not able to compare their children’s behavior with their peer group in the same setting (Rescorla et al., 2012). These findings are in line with those of other researchers (e.g., Heyman et al., 2018), who have found that parents rate their preschool children’s social skills higher than do their teachers, and these differences have also been associated with the socioeconomic status of families. Hence, teachers’ bias as well as the parents’ tendency to provide more positive evaluations of their child could be important aspects to consider when evaluating children from families of low socioeconomic status. Keeping this in mind, the research question of whether preschool children’s learning outcomes could be explained by their social emotional competence, behavioral difficulties, and demographic variables was posed.

### Correlations of Preschool Children’s Social Emotional Competence and Their Social, Emotional, and Behavioral Difficulties in Evaluations of Their Teachers and Parents

A separate research question was devoted to the association between the social emotional competence and adjustment of preschool children. In line with the findings of previous research (e.g., Raver and Knitzer, 2002; Merrell and Guelder, 2010; Panadero and Alonso-Tapia, 2013; Panadero et al., 2017), it was expected that the social emotional competence of preschool children would be negatively correlated with their emotional and behavioral difficulties, and positively correlated with their prosocial behavior. The results of the current research were in line with these findings, especially for teachers’ evaluations. In the teacher reports, a higher evaluation in SSIS SEL social emotional competence subscales (self-awareness, self-management, social awareness, relationship skills, and ability to make responsible decisions) of the preschool children was associated with lower ratings of all difficulties and higher ratings of prosocial behavior in SDQ. However, in parents’ evaluations, just two domains of social emotional competence—self-awareness and self-management—were significantly related to less difficulties (emotional, conduct, hyperactivity, and peer problems), and more prosocial behavior of their preschool children. According to parents, higher evaluations of the children’s relationship skills were associated with lower ratings of their emotional problems in SDQ. In this study, the parents’ reports of strengths and difficulties of their children were not associated with their children’s social emotional skills such as responsible decision making and social awareness. A similar trend can be observed for the reports of one domain of the social emotional competence—relationship skills. Thus, one could conclude that parental evaluations of their preschool children’s better interpersonal skills are related to lower ratings of the children’s social, emotional, and behavioral difficulties. These slight differences in the correlations in the two informants’ reports could be associated with the different teacher/parent-child relationship contexts. Parents are more aware of the individual aspects of their children’s social emotional competence and their ability to understand themselves and regulate their emotions, thoughts, and behavior. It is positive that parents notice these important skills in their children. In turn, teachers are more able to base their evaluations on direct observations in the wider classroom context (e.g., Winsler and Wallace, 2002; Van der Ende et al., 2012). Sometimes, when observing a child’s individual contacts with adults, parents, or specialists, the behavior of a child may seem adaptive, but observing it in the social context available to the teacher could add valuable evidence of the child’s ability to interact with peers and adults, and to cooperate in a group. This suggests the necessity for specialists to consider the perspective of multiple evaluators to generate a more complete picture of children’s developmental patterns (Stone et al., 2010; Fält et al., 2018).

### The Role of Social Emotional Competence and Difficulties in Teacher-Reported Learning Outcomes of Preschool Children, Considering Parental Socioeconomic Status and Vulnerability Status of a Child

It was found that according to teachers, better social emotional competence and prosocial behavior both have a significant role in preschool children’s academic motivation, learning engagement, and academic performance. Higher social, emotional, and behavioral difficulties significantly undermine these learning outcomes.

This study found that parents with higher education (tertiary) and/or occupation rate their children as having higher social emotional competence and prosocial behavior, and lower behavioral difficulties compared to parents with lower socioeconomic statuses. The teachers rated advantaged students higher in social emotional competence and lower in behavioral difficulties compared to their disadvantaged peers or those at risk of social exclusion (e.g., those with learning disabilities or from a minority group). However, parental education and occupation as well as a child’s gender and belonging to the vulnerable group were not significantly associated with learning outcomes in this sample. Nevertheless, the social emotional competence in parallel with social, emotional, and behavioral problems remains associated with learning outcomes when parents’ education, occupation, and child’s vulnerability were controlled. Considering the diverse family backgrounds of students represented in the class or group, the social emotional competence can be perceived as a significant resource which allows to balance the effect of student family inequalities. Thus, it is important to facilitate young children’s social emotional development to promote inclusion and equal opportunities for all children. This conclusion has been supported by other researchers (e.g., Fantuzzo et al., 2003; Blair and Diamond, 2008; Domínguez and Greenfield, 2009; Bulotsky-Shearer et al., 2012).

### Social Emotional Competence as a Mediator

It was found that according to the teachers’ evaluations, the preschool children’s social emotional competence mediates the association between their emotional and behavioral difficulties and learning outcomes. This indicates that both lower levels of difficulties and higher levels of social emotional competence are essential for the enhancement of learning motivation, engagement, and performance among preschool children. Considering the possibility of the reciprocal relationship, it is crucial to recognize the mediating role of social emotional competence in relation between learning outcomes and behavioral difficulties. Social emotional competence could serve as a significant resource for children, including those with behavioral difficulties, to maintain satisfactory learning activity. This provides a support for the necessity to include social emotional learning as a resource for both, promoting learning outcomes and managing of difficulties.

Understanding the role of social emotional competence in learning is of special importance in the Latvian context, in which the focus in preschool education is on preparing children for school. Parents and teachers often emphasize children’s progress in reading, writing, and arithmetic, as well as the importance of positive behavior and academic motivation without paying proper attention to a purposeful investment in the social emotional development of children. Evidence that social emotional competence is a key factor in healthy development, academic engagement, and successful learning strongly supports the importance of social emotional learning in early education. As children’s social emotional development during preschool is rapid, an early universal intervention promoting social emotional learning and resilience would release children’s resources for cognitive work and learning. Other researchers have also proposed the importance of integrating the promotion of social emotional learning and resilience, as well as approaches to prevent social, emotional, and behavioral problems in one intervention model (Cavioni et al., 2020). Moreover, it is known that early intervention is more effective, and thus, a preschool setting is optimal to start developing children’s social emotional competence systematically, since it is one of the key elements for ensuring protection and better mental health, inclusion, and successful learning.

### Implications, Limitations, and Recommendations

The findings of this study highlight the importance of universal social emotional learning in preschool children for promoting children’s social emotional development and academic engagement, and preparing them for academic learning in primary school. Thus, the early development of social emotional competence serves as a medium of preparation for primary school. In addition, curricula must focus on enhancing the social emotional learning of children from more deprived backgrounds or who are vulnerable to exclusion, thus reducing inequality.

This study emphasizes the value of the multi-informant approach for the assessment of young children. Comparison of different perspectives can be beneficial for a better understanding of children’s social emotional competence, strengths and difficulties, and learning outcomes, since preschool children are not able to complete self-report questionnaires, and neither are there any objective measures (i.e., grades) to evaluate their outcomes. On the other hand, more research may be undertaken to develop innovative and creative tools which help young children to express their views on their behavior and learning.

This study also has some limitations. The sample for this research included almost all preschool children from one Latvian region, and hence, does not provide geographical representativeness of the whole Latvian preschool children population. However, the Sigulda region does not have any differentiating factors that set it apart from other regions of Latvia. Another limitation is that the teachers’ and parents’ evaluations are indirect measures of the child’s true social emotional competence and social, emotional, and behavioral difficulties. The answers received by the survey method could be biased, especially when competencies and outcomes are evaluated simultaneously while addressing the vulnerability status of the respondents. The current research did not include direct observations of the children’s social emotional skills, and emotional and behavioral difficulties. By conducting inter-rater reliability, this limitation can be alleviated; thus, the multi-informant (parent-teacher or two teachers) approach was used. Nevertheless, even a consistency among the teacher and parents’ evaluations does not obviate a lack of direct observations. Furthermore, in this research, we tried to overcome the obstacle that the learning outcomes of preschool children cannot be assessed based on their grades. Despite the very good internal consistency of the developed three-item measure, a more robust instrument should be applied to explore preschool children’s learning outcomes.

## Data Availability Statement

The raw data supporting the conclusions of this article will be made available by the authors upon request.

## Ethics Statement

The studies involving human participants were reviewed and approved by the the Ethics Committee for Humanities and Social Sciences of the University of Latvia on 12 December, 2019. Before they completed the survey, respondents gave informed consent to participate. Written informed consent was provided by the children’s legal guardian/next of kin for the use of the survey data in this publication.

## Author Contributions

BM: leading writer, arranging the research in Latvia, collecting of data. ISu and ID: collecting of data, contributing to writing. ISt: contributing to writing. CC and IG: key contribution to designing the research and revising the manuscript. LC: data analyses and contributing to writing. PB: revising the manuscript. MO’R: collecting of data and preparing the files for data analysis. All authors contributed to the article and approved the submitted version.

## Conflict of Interest

The authors declare that the research was conducted in the absence of any commercial or financial relationships that could be construed as a potential conflict of interest.

## Publisher’s Note

All claims expressed in this article are solely those of the authors and do not necessarily represent those of their affiliated organizations, or those of the publisher, the editors and the reviewers. Any product that may be evaluated in this article, or claim that may be made by its manufacturer, is not guaranteed or endorsed by the publisher.
